# Smells like … no evidence that odors influence the attentional blink

**DOI:** 10.3758/s13414-024-02986-4

**Published:** 2024-12-19

**Authors:** Ryan Hackländer, Pamela Baess, Christina Bermeitinger

**Affiliations:** Cognitive Psychology, Institute of Psychology, University of Hildesheim, Universitaetsplatz 1, D–31141 Hildesheim, Germany

**Keywords:** Olfaction, Attention, Attentional blink, Lavender, Peppermint

## Abstract

The attentional blink (AB) paradigm is frequently used to investigate temporal attention. Essentially, rapid serial visual streams of several distractors and two targets are presented. The accuracy in detecting the second target stimulus (T2) decreases in the time window between 100 and 500 ms following accurate detection of the first target stimulus (T1). In two experiments, Colzato et al. *Attention, Perception, & Psychophysics*, *76*, 1510–1515, (2014) reported evidence for a modulation of the AB effect depending on the presentation of different ambient odors: Peppermint increased the AB compared with lavender. In the current study, we tried to replicate their basic findings while using different methods and procedures to present the lavender versus peppermint odorants. In three experiments, we found no evidence that these odorants influence the AB effect. We discuss our findings in comparison with those from Colzato et al., in relation to other empirical research in this field as well as in regard to different hypotheses concerning how odorants may influence human cognition.

After decades of being mostly ignored by psychological science, olfaction has begun to garner interest as a worthy topic of investigation over the past several years. In particular, researchers have been increasingly interested in how nonolfactory stimuli affect olfactory perception and how olfaction influences thoughts and behaviors, a field of research termed olfactory cognition. The increasing interest in olfactory cognition seems to be driven by theoretical interest in multimodal perspectives of perception and cognition (e.g., Ho & Spence, 2005) as well as an increased interest in the potential practical benefits of the use of odors, such as in aromatherapy (e.g., Cooke & Ernst, 2000). Given the novelty of this research area, it follows that various perspectives have arisen concerning how olfaction influences cognition and which cognitive processes are influenced by odors.

In 2014, a study by Colzato and colleagues found evidence that specific odorants can influence temporal attention. The researchers utilized the attentional blink (AB) paradigm to measure temporal attention. In this paradigm, subjects view a rapid serial visual presentation (RSVP) consisting of a number of distractors and two targets. The typical finding, referred to as the AB effect, is that subjects have difficulty reporting the identity of the second target when it is presented shortly after (usually in a window ranging from 100 to 500 ms) the first target (for reviews, see Dux & Marois, 2009; Martens et al., 2002; Martens & Wyble, 2010; Shapiro, Arnell, et al., 1997a).

Previous research has shown that the size of the AB effect can be influenced by factors such as personality or interindividual differences (e.g., McLean & Arnell, 2010; Willems & Martens, 2016), relevance/salience of the second target (e.g., own name/emotional/threatening/angry stimuli, e.g., Maratos et al., 2008; Shapiro, Caldwell, et al., 1997b; Trippe et al., 2007), semantic relations between the target words (e.g., Maki et al., 1997), current mood (e.g., Olivers & Nieuwenhuis 2006; Vermeulen, 2010), and focus of attention (e.g., Olivers & Nieuwenhuis, 2005). Specifically, Olivers and Nieuwenhuis (2005, 2006) found that subjects who performed the task in a more distributed mental state showed an attenuated AB effect. This attenuation was used to support the authors’ claim that subjects generally overinvest resources in identifying T1 and therefore do not have enough attentional resources to identify T2 if it appears too quickly following T1.

Accordingly, Colzato and colleagues (2014) aimed to determine if specific odors could influence the size of the AB effect as there are odors which are associated with different arousal states. Specifically, they hypothesized that peppermint, an odor typically associated with arousal and more focused attention (especially in difficult conditions, e.g., Ho & Spence, 2005), would increase the size of the AB effect, compared with lavender, an odor typically associated with calmness, relaxation, and a broader focus of attention (e.g., Basevitch et al., 2011). Across two experiments the authors found support for their hypothesis and argued that “the AB was less pronounced when participants were exposed to the more relaxing aroma” (p. 1514).

Despite the consistency of their findings across two experiments, there are several important questions concerning both the reported effect and the study itself. First, it is unfortunate that the authors did not reveal how they calculated the AB effect and if this was the same across both experiments. This is crucial information which would allow future researchers to more accurately compare results. Second, it is not clear whether the authors attribute the changing effect to the peppermint or the lavender condition. In their introduction, they focus on peppermint as the odor that influences the change (i.e., increase) of the AB effect. In contrast, in the discussion, lavender then seems to be responsible for the change (i.e., decrease) of the AB effect. In their Experiment 1, peppermint seemed to increase the AB effect, while the lavender group showed an AB effect similar to that of the control group. Based on the literature cited (e.g., Olivers & Nieuwenhuis, 2005) and with reference to the overinvestment hypothesis, one would expect a reduction in the AB effect to occur with broader attentional focus (i.e., lavender), but not necessarily an increase in the AB effect to occur in conditions where the attentional focus is narrowed. Additionally, it is not clear that a more narrowed focus, as modulated by peppermint odor, would necessarily mean that attention is more narrowly focused on what the authors consider to be the main task. Indeed, attention may become more focused, but on something other than the main task. Importantly, this point is not necessarily specific to the experiment by Colzato and colleagues (2014), but is more general to any research interested in modulating the AB effect by increasing attentional focus. Third, Colzato and colleagues (2014) did not expound upon the theoretical bases for odorants or odors producing differences in the AB effect.

With reference to the third point, odorants theoretically have several methods of influencing human cognition, including pharmacologically, psychologically, or through tactile stimulation (for reviews, see Herz, 2009; Johnson, 2011). A pharmacological explanation would mean that the specific chemicals that compose an odorant bind to receptors in the brain and have specific effects (similar to any other drug effect). This type of effect would require the chemicals to cross the blood brain barrier, which reportedly takes around 20 min (Herz, 2009). Given that each experimental session in the studies reported by Colzato and colleagues (2014) lasted about 10 min, it is unlikely that there were any pharmacological effects of odorants on attention.

Odors may also have psychological effects on human cognition. First, the presence of an odor may either increase or decrease (in terms of pleasantness) an individual’s subjective mood (for a review, e.g., Herz, 2002). Given that mood seems to be something that changes rather slowly, as opposed to the experience of a sudden emotion (e.g., Beedie et al., 2003), these types of effects would theoretically develop over time. As it relates to the current experiments, such mood effects would also rely on differential effects on mood between the two odors. Although Colzato and colleagues (2014) found no evidence of this being the case, they indicated that explicit measures of mood may not be sensitive enough to find such differences.

In addition to effects on mood, there could be effects on attention based on experience and associative learning between odors and more specific events. Expectancy effects refer to the presence of an odor, which is consciously perceived and identified, leading to the conscious expectation of a certain emotion. Such expectancies would be due to past experiences. For example, if someone recognizes lavender, it could retrieve an episodic memory of a calming environment in which lavender was present, such as during a massage. This episodic memory could potentially influence mood or more directly influence the focus of attention. Similarly, associative effects refer to an odor leading to the priming of a specific association with the odor.

As opposed to expectancy effects, associative effects do not necessarily involve the retrieval of an episodic memory, and therefore the priming could be outside the conscious awareness of the individual. Although it is not exactly clear, it is reasonable to assume that expectancy and associative effects should take place shortly following perception, and certainly would require shorter periods for effects to take place than mood effects. Last but not least, it might be that the odors themselves differentially distract attention. For example, the odors may differentially trigger subjects’ search for the source and/or identity of the odor (i.e., this can be seen as an additional task leading to distraction of attention, similarly see also Olivers & Nieuwenhuis, 2005).

A third possibility is that the odorants could have had a tactile, rather than olfactory, influence on cognition. Certain odorants activate not only the olfactory nerve, but also the trigeminal nerve, which is what leads to tingling or temperature sensations associated with some odorants (such as peppermint/menthol; e.g., Doty et al., 1978). When the inhalation of odorants activates the trigeminal nerve, it is possible for this nonolfactory activation to directly influence patterns of neural firing, cognition, and behavior. For example, Stuck et al. (2007) presented sleeping subjects with either an olfactory activating stimulus (which did not activate the trigeminal nerve) or a trigeminal activating stimulus (CO2), which did not activate the olfactory system. The researchers found that the presentation of the trigeminal activators led to increased markers of arousal during sleep, while the olfactory activating stimuli did not. Supporting this, other research has also shown that trigeminal activating odorants and chemical compounds can influence both neural firing as well as behavior (Boyle et al., 2007; Lundström et al., 2005; Stuck et al., 2011). If lavender and peppermint differentially activate the trigeminal nerve it could be that this tactile stimulation influenced attention. This could potentially explain why lavender showed similar effects to the control group (i.e., no difference between the two groups in trigeminal activation), while peppermint showed a larger AB effect (i.e., larger trigeminal activation). Although the question of whether trigeminal activation is/can be responsible for ensuing behavioral effects is interesting, it is outside the scope of the experiments of the current report and therefore will not be discussed further.

As mentioned before, the article by Colzato et al. (2014) leaves some important questions open regarding the nature of the observed effect. Thus, in the current paper, we report three different experiments that test how peppermint and lavender influence the AB effect. Our specific aims were (a) to determine whether we could verify the finding that peppermint leads to a larger AB effect than lavender (Colzato et al., 2014) with an exact conceptual replication (see Petty, 2018; for a discussion of different types of replications, see, e.g., Derksen & Morawski, 2022; LeBel, 2015; Oberauer & Lewandowski, 2019; Zwaan et al., 2018) while presenting the odorants via an olfactometer (i.e., instead of an ambient presentation as used by Colzato et al., 2014), (b) to examine the generalizability of the differential AB effect with different odorant presentation methods, (c) to determine how long of an exposure time is required to produce said effects, and (d) to determine whether the effect builds with exposure over time or whether the effect reaches its apex shortly following perception. In order to make our research as transparent as possible, we describe the presentation of the odorants and in general our procedure and material as detailed as possible. Furthermore, we used a common AB effect measure (see Design and Statistical Analyses section for the calculation).

## General method

All of our experiments were designed to conform as closely as possible to the experiments of Colzato et al. (2014), with the obvious main difference being the method of presentation of the odorants (i.e., direct via an olfactometer rather than ambient). Methods common to the three experiments are presented here. Methods unique to each experiment are described in the corresponding sections below.

### Materials and procedure

All experiments were programmed and run using E-Prime 2.0 software (Schneider et al., 2002). All visual stimuli were presented on a 17-in. CRT monitor with the refresh rate set to 75 Hz. Instructions were presented on the screen.

### Olfactory presentation

Odorants were presented directly to subjects with the aid of an olfactometer (OG001, Burghart Messtechnik, Germany). This model is a discrete-presentation olfactometer, which presents odiferous air at discrete times, rather than having a continuous flow of air. The odorants were transmitted from jars to the subjects through tubes that ended about 10 cm below the subject’s nose. In between the end of the tube and the subject’s nose there was a filter which ensured that no tactale stimulation was felt from the air puff, while still allowing for dispersal of the odorant (and perception of the odor).

The odorants were purchased commercially from Primavera Life (Germany). The specific odorants were lavender (Lavendel fein) and peppermint (Pfefferminze). “Lavendel fein” was extracted from Lavandula angustifolia and is composed of chemotypes Geraniol, Limonen, and Linalool. “Pfefferminze” was extracted from Mentha × piperita and is composed of chemotypes Menthol and Menthone. Odorants were deposited onto an odorless cotton pad and placed into the presentation jars of the olfactometer. Based on pretests aimed at determining what amount of material would lead to roughly equal subjective experience of odor intensity we used 50 drops of lavender and five drops of peppermint. The odorants were replaced frequently to maintain freshness.

### AB task, and procedure of the AB task and the olfactory presentation

**Fig. 1:**
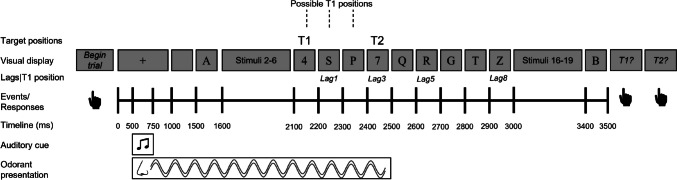
Visualization of the AB procedure used across all three experiments. Note that T2 in the figure is an example of a trial with lag = 3 and T1 as the seventh stimulus. Similarly, the lag positions shown in the figure refer specifically to trials where T1 is the seventh stimulus. If T1 were at a different position, then so would the possible lag positions. Note also that the distance on the timeline does not directly correspond to the amount of time that has passed

The AB task was based on that utilized by Colzato et al. (2014; for a visualization, see Fig. 1). Subjects were presented with an RSVP consisting of 18 distractors and two targets. Distractors were letters and targets were digits; both were presented in Times New Roman 16-pt font. As in the study by Colzato et al. (2014), the fixation cross and all stimuli were presented in the middle of the screen in black on a gray background. The distractor stimuli (capital letters) were chosen randomly, with no doubles, on each trial from the standard American alphabet (excluding the letters *I* and *O*). The target stimuli (digits) were chosen randomly, on each trial from the Latinized Hindu-Arabic numerals 2 to 9. The subjects’ task was to report the identity of the digit targets (T1 and T2) following the serial presentation. The subject started each trial self-paced by pressing the space bar. Each trial began with the presentation of a fixation cross, which remained on the screen for 1,000 ms. After 500 ms following the onset of the fixation cross, there was (a) the onset of an auditory cue (a 500 Hz sinus wave tone) lasting 250 ms, which instructed subjects to inhale, and (b) the presentation of the odorant (on trials when odorants were presented), which involved a 2,000-ms puff of air. Following the fixation cross there was a 500-ms blank screen. This blank screen was immediately followed by the serial presentation of distractors and targets. Each of the 20 stimuli in the RSVP stream was presented for 66.67 ms with a 33.33-ms blank screen presented between stimuli (i.e., their SOA was 100 ms). Subsequent to the serial presentation subjects were presented with a screen asking them to report the identity of one of the targets. Following a response, subjects were presented with a second response screen asking them to report the identity of the other target. In line with the method used by Colzato et al. (2014), the order of the presentation/responses was not considered when calculating report accuracy. The serial position of T1 varied randomly between positions 7, 8, and 9. T2 was presented immediately following T1 (= Lag 1) or following the presentation of 2, 4, or 7 distractors (i.e., Lags 3, 5, and 8, respectively). The number of trials in each block and the number of blocks depended on the individual experiment.

### Discrimination and odor rating task

Following the AB task, subjects performed an olfactory discrimination task to ensure they could discriminate between peppermint and lavender scents. In this task, subjects were presented with the two odorants, by means of the olfactometer, on each trial, and they had to decide whether the two were the same as, or different from, one another. Subjects were instructed to inhale when they heard an auditory cue. Each trial began with an auditory cue (500 Hz sinus wave tone) being presented for 500 ms. The onset of the tone was simultaneous with the presentation of the first odorant, with a puff lasting 2,000 ms. After 5,500 ms after the offset of the first auditory cue, the second auditory cue (same as first) and the second odorant were presented. After 5,500 ms after the offset of the second auditory cue, a response screen appeared requiring the participants to decide if both odorants were the same or different. The discrimination task consisted of 16 trials (eight same and eight different) in randomized order. On half of the trials, peppermint was presented as the first odorant, and on the other half of the trials, lavender was presented as the first odorant.

Immediately following the discrimination task, subjects performed an olfactory rating task. This task consisted of subjects being presented with the two odorants (peppermint and lavender), again by means of the olfactometer, in random order and being asked to rate them in terms of intensity, familiarity, and pleasantness on scales ranging from 1 (*not intense/familiar/pleasant*) to 9 (*very intense/familiar/pleasant*).

Given that discrimination performance and subjective odor ratings were not deemed as critical to the understanding of the main investigation (i.e., the AB), the data are presented only in appendices. The discrimination data from the three experiments can be found in Appendix 1 Fig. 9, while data from the subjective odor rating task can be found in Appendix 2. The most important takeaways are that the discrimination rate was significantly above chance for all three experiments, and there were no differences in subjective ratings between lavender and peppermint in any of the three experiments, with the one exception being that peppermint was rated as more familiar and pleasant than lavender in Experiment 3 only. One further finding was that discrimination rates differed between the three experiments, but given that our manipulations for each experiment were all within subjects, we believe this finding to have little relevance for the understanding of our main findings.

### Design and statistical analyses

For each experiment we were interested in three different dependent variables. The first was the T1 accuracy. The second main dependent variable was T2│T1, which is the report accuracy of T2 on trials when T1 was correctly reported. In line with the recommendations on AB interpretation (MacLean & Arnell, 2012), the interaction between the factors odor (peppermint, lavender) and lag (Lag 1, Lag 3, Lag 5, Lag 8) would be indicative of a difference in the AB based on different odors. We further calculated the AB effect using the following formula: AB effect = Lag 5 (T2│T1) − Lag 3 (T2│T1). Although the AB effect is not independent of the second dependent variable, T2│T1, we decided a priori to include it as a dependent variable, regardless of whether there was a main effect of lag in the overall ANOVA on T2 performance or an interaction between the factors odor and lag, given the importance of this measurement for comparisons with the report by Colzato et al. (2014).^1^ For T1 and T2|T1 accuracy, an ANOVA with the within-subject factors odor (lavender vs. peppermint) and lag (Lag 1, Lag 3, Lag 5, Lag 8) was performed.

### Ethical considerations

All subjects participated of their own free will and were notified that they could end their participation at any time without risk of penalty. All subjects were informed of any potential risks before signing informed consent. Furthermore, any subjects reporting odor allergies were forbidden from participation and were informed of other experiments in which they could participate. All subjects were students of the University of Hildesheim and received partial course credit or monetary remuneration for their participation. Finally, all studies were performed with approval of the Local Ethical Committee of the Faculty 1 at the University of Hildesheim and were done in accordance with the current version of the Declaration of Helsinki (World Medical Association, 2024).

### Sample-size calculations

Colzato et al. (2014) reported medium to large effect sizes for the modulation of the AB effect by odor across their two experiments. Using these effect sizes as a guide, we calculated an estimated required sample size to find medium to large modulations of the AB effect by odor. With the aid of G*Power 3 (Faul et al., 2007) we performed an a priori sample size calculation using a one-tailed paired-samples *t* test with an alpha error of .05 and a beta error of .2. For an effect size of *d*_z_ = .5, we would require a sample of *n* = 27. Accordingly, we aimed to collect a sample of at least 27 subjects in each of the three experiments.

## Experiment 1

### Method

#### Subjects

Thirty-seven subjects participated in this study. Three subjects were removed from the analyses due to technical errors with the olfactometer. The final sample (*n* = 34) had a mean age of 21.79 years (*SD* = 4.5) and consisted of 30 women and four men. All subjects reported average or above-average olfactory capabilities and normal or corrected-to-normal vision.

#### Material, procedure, and design

The method for the AB task followed exactly the procedure described in the general method section for each trial, with a randomized, trial-wise presentation of either lavender or peppermint. Subjects performed two blocks each consisting of 144 trials (72 with lavender), leading to a total of 288 total trials. Each experimental session lasted approximately 90 min. We used a 2 (odor: lavender vs. peppermint) × 4 (lag: 1, 3, 5, 8) design, with all factors varied trial-by-trial within subjects.

#### Hypothesis

Based on the findings from Colzato et al. (2014), we would expect an interaction between odor and lag for T2|T1 accuracy. More specifically, we would predict a larger AB effect in the lavender than the peppermint condition.

### Results

**Fig. 2:**
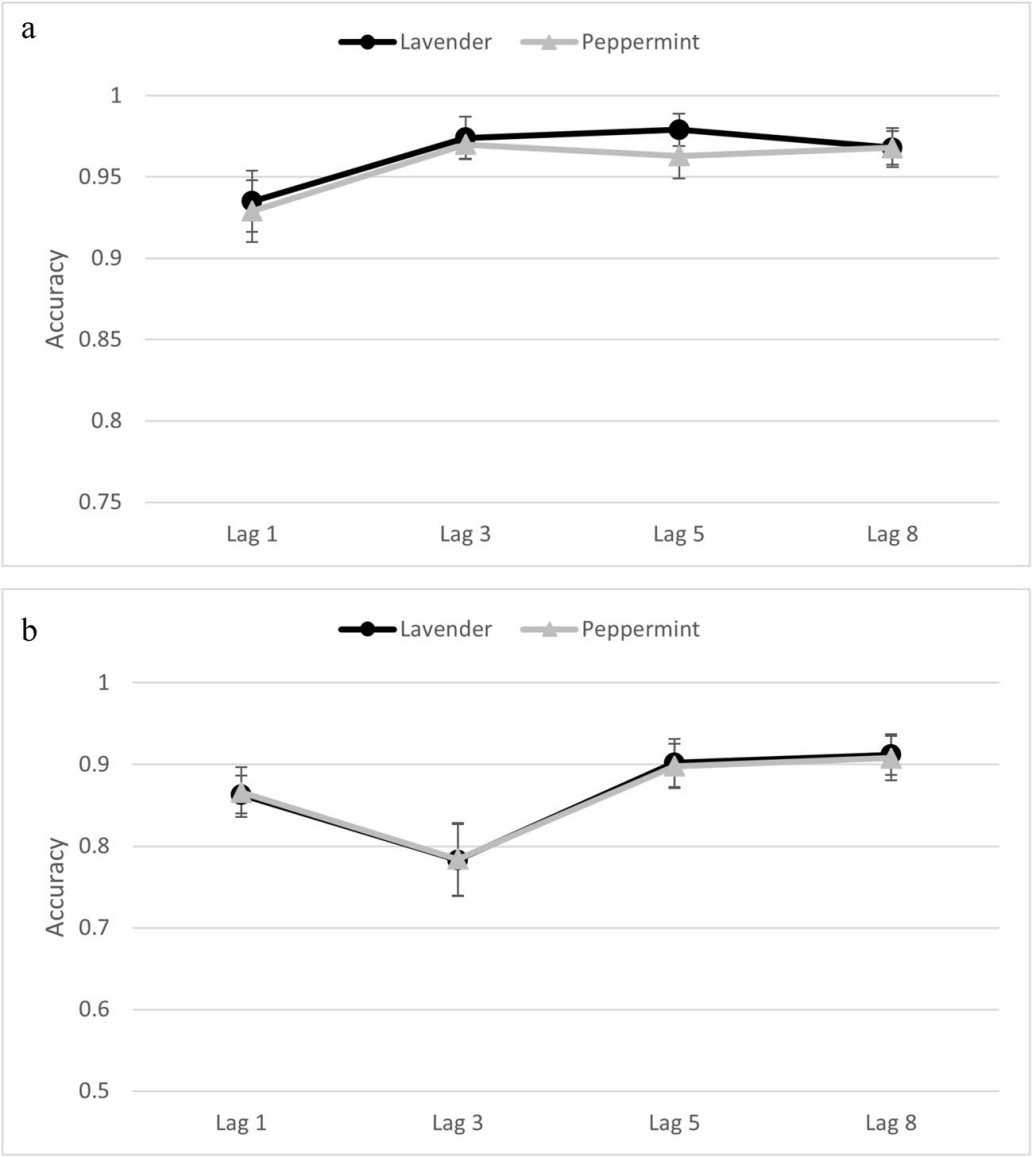
**a** Reporting accuracy for T1 as a function of odor and lag in Experiment 1. Error bars represent 95% confidence intervals. **b** Reporting accuracy for T2│T1 as a function of odor and lag in Experiment 1. Error bars represent 95% confidence intervals

Accuracy data from T1 and T2│T1 (see Fig. 2a & b, respectively) were submitted to separate repeated-measures analyses of variance (ANOVAs) with the independent variables odor and lag.

#### T1

The ANOVA with T1 accuracy revealed a significant main effect of lag, *F*(3,99) = 21.20, *p* < .001, ${\eta }_{p}^{2}$=.39. Pairwise comparisons, with Bonferroni corrected critical *p* values = .0167, revealed that T1 detection was lower when followed by Lag 1 (*M* = .93) than all other lags (Lag 3: *M* = .97, Lag 5: *M* = .97, Lag 8: *M* = .97), all *p* values < .001. There was no difference between Lag 3, Lag 5, or Lag 8, respectively, all *p *values > .471. No other main effects or interactions were significant, all *F* values < 1.85, all *p* values > .180.

#### T2│T1

The ANOVA revealed a significant main effect of lag, *F*(3,99) = 24.32, *p* < .001, ${\eta }_{p}^{2}$ =.42. Pairwise comparisons, with Bonferroni corrected critical *p* values = .0167, revealed that T2|T1 detection was lower at Lag 3 (*M* = .78), than at Lag 1 (*M* = .87), Lag 5 (*M* = .90), and Lag 8 (*M* = .91), all *p* values < .001. T2|T1 detection was also significantly lower at Lag 1 than at Lag 8, *p* < .001. No other differences in lags were significant, all *p* values > .019. There were no other significant effects (all *p* values > .865), most importantly, there was no significant interaction between odor and lag, *F*(3,99) = 0.05, *p* = .984, ${\eta }_{p}^{2}$ < .01, which would have indicated a difference of the AB depending on odor.

#### AB effect

The AB effects for peppermint and lavender were assessed as the difference in T2|T1 accuracy between Lag 5 and Lag 3 (peppermint: *M* = .11, 95% CI [.07, .15], lavender: *M* = .12, 95% CI [.07, .16]). The difference between peppermint and lavender was clearly not significant, paired *t* test: *t*(33) = .24, *p* = .809. However a one sample *t* test (*df* = 33) against 0 showed that the overall AB effect (*t* = 6.23, *p* < .001) was significant.

### Discussion

In Experiment 1, we performed a conceptual replication of the experiments reported in Colzato et al. (2014). Subjects performed an AB task while being directly presented with lavender and peppermint odorants by means of an olfactometer. The odorants varied randomly on a trial-by-trial basis. Colzato and colleagues reported an accentuation of the AB effect in the presence of peppermint odor compared with lavender; however, we failed to find any indication that the AB effect was modulated by these odorants, though the AB effect itself was clearly obvious in our data.

Given that we were unable to replicate the findings of Colzato and colleagues (2014), we focused on the differences in our experiments that may have accounted for the disparate findings. The most obvious difference between the experiments lies in the delivery method of the odorants. In their study, Colzato and her team presented odorants ambiently. In our study, we presented odorants directly to the subjects by means of an olfactometer. Although this difference is superficially large, it is theoretically unclear why a difference in delivery method should influence how the odorants modulate the AB effect. Indeed, other studies in the field of olfactory cognition have found comparable effects regardless of whether odor presentation was ambient or direct (e.g., Hackländer & Bermeitinger, 2017), and other studies have found AB effects with direct presentation of odors by means of an olfactometer (Robinson et al., 2013).

Thus, rather than focusing on the differences in odorant delivery method, we decided to investigate the time of exposure differences. Across the two experiments of Colzato et al. (2014), subjects were exposed to only one odorant throughout the entire experimental procedure. In our studies, the odorants shifted on a trial-by-trial basis. Since the odorant was presented directly before the onset of the RSVP, it could be that the odor did not have enough time to influence cognition. This hypothesis seems even more plausible than that of different presentation methods given that odor sensation and perception takes longer than sensation and perception in the other senses (Herz & Engen, 1996).

Therefore, in Experiment 2, we aimed to test the hypothesis that olfactory modulation of the AB effect is dependent on a certain amount of time passing for an odor to be perceived and consequently to alter attention. To achieve this test, we performed a near replication of our Experiment 1 with one exception. Following each odor-trial, in which an odorant was presented before the onset of the RSVP, subjects completed a no-odor trial. On no-odor trials, subjects viewed the RSVP without the introduction of an odorant before the trial. This allowed us to test whether the presentation of an odorant would have an effect on the AB either immediately following the presentation of the odorant, or after a delay of several seconds (which is more than enough time to allow the odor to be sensed and perceived; Olofsson, 2014).

## Experiment 2

### Method

#### Subjects

Forty-one subjects participated in this study. Two subjects were removed from the analyses due to technical errors with the olfactometer. The final sample (*n* = 39) had a mean age of 21.97 years (*SD* = 3.4) and consisted of 32 women and seven men. All subjects, except for one, reported average or above average olfactory capabilities. The subject who reported below-average olfactory capabilities was found to perform at an average rate in the discrimination task, in terms of both accuracy and RT, and was therefore not removed from the data set. All subjects, except for two, reported normal or corrected-to-normal vision. The two subjects who reported requiring, but not wearing, visual aids performed numerically better (in terms of both T1 and T2 detection) than other subjects. Based on their (at least) average performance, we decided against removing them from the data set.

#### Material, procedure, and design

The method for Experiment 2 was identical to that of Experiment 1, except for the following differences. (1) After each odor trial (the order of peppermint and lavender again was determined randomly), there was a no-odor trial in which neither an odorant nor an air puff was presented. Even in no-odor trials, a tone was presented which cued subjects to inhale. (2) In order to ensure the same amount of odor trials between Experiment 1 and 2, subjects participated in four blocks with 144 trials each, leading to 576 total trials per subject (288 with an odorant). In each block there were 36 trials with lavender odorant, 36 trials with peppermint odorant, and 72 trials with no odorant (and no air puff) presented (36 after a lavender trial, 36 after a peppermint trial). Each experimental session lasted approximately 120 min.

In addition to the standard factors of odor and lag, this experiment included the factor of trial type, referring to whether an odorant was presented on the trial (odor trial) or not (no-odor trial). This resulted in a 2 (odor: lavender vs. peppermint) × 4 (lag: 1, 3, 5, 8) × 2 (trial type: odor vs. no-odor) design, with all factors varied within subjects.

#### Hypotheses

Based on the findings from Colzato et al. (2014), we would expect an interaction between odor and lag for T2|T1 accuracy. More specifically, we would predict a larger AB effect in the lavender than the peppermint condition. Alternatively, if odor requires a certain amount of time to have an effect on attention, we would expect an interaction between odor, lag, and trial type for T2|T1 accuracy. More specifically, we would predict a larger AB effect in the lavender than the peppermint condition, but only on no-odor trials.

### Results

**Fig. 3:**
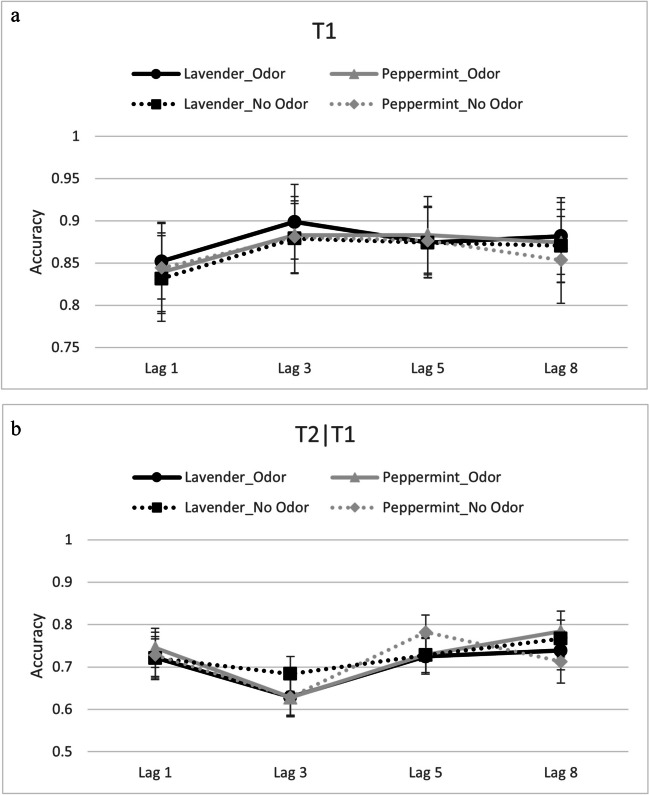
**a** Reporting accuracy for T1 reporting accuracy as a function of odor, trial type, and lag in Experiment 2. Error bars depict 95% confidence intervals. **b** Reporting accuracy for T2│T1 reporting accuracy as a function of odor, trial type, and lag in Experiment 2. Error bars depict 95% confidence intervals

Accuracy data from T1 and T2│T1 (see Fig. 3a & b, respectively) were submitted to separate repeated-measures ANOVAs with the independent variables of odor, lag, and trial type.

#### T1

The ANOVA revealed two significant main effects. The main effect of trial type, *F*(1,38) = 4.75, *p* =.036, ${\eta }_{p}^{2}$ =.11, was significant whereby subjects reported T1 more accurately on odor trials (*M* =.87) than on no-odor trials (*M* =.86). Moreover, the main effect of lag, *F*(3,114) = 16.57, *p* <.001, ${\eta }_{p}^{2}$ =.30, was significant as well. Pairwise comparisons, with Bonferroni-corrected critical *p* values = .0167, revealed that T1 accuracy was lowest at Lag 1 (*M* =.84) compared with all other lags (Lag 3: *M* = .89, Lag 5: *M* = .88, Lag 8: *M* = .87), all *p* values < .001. Furthermore, accuracy of T1 was better when T2 was at Lag 3 than at Lag 8, *p* = .005.

#### T2│T1

The ANOVA revealed only a significant main effect of lag, *F*(3,114) = 11.13, *p* < .001, ${\eta }_{p}^{2}$ =.23. Pairwise comparisons, with Bonferroni-corrected critical *p* values = .0167, revealed a typical AB pattern (i.e., T2|T1 detection at Lag 3 was worse compared with all other lags, all *p* values < .003). There were no other significant effects (all *p* values > .332), most importantly, the interaction between odor and lag, indicative of a modulation of the AB effect based on odors, was clearly nonsignificant, *p* = .285.

#### AB effect

The AB effect was submitted to an odor (lavender vs. peppermint) × trial type (odor vs. no-odor) repeated-measures ANOVA. Neither the main effects, nor the interaction, were significant, all *F *values < 3.10, all *p* values > .08. However and in accordance with the T2 | T1 analysis, the intercept was significant, *F*(1,38) = 22.01, *p* < .001, ${\eta }_{p}^{2}$ =.37, indicating an overall AB effect (independent of odor or type of trial).

### Discussion

In Experiment 2 we again found no evidence that odors differentially modulate the AB effect. Experiment 2 differed from Experiment 1 in one major detail: The introduction of no-odor trials allowed us to determine whether odors needed a certain amount of time to be perceived before they could alter temporal attention. We again could not replicate the findings of Colzato and colleagues (2014), regarding the differential effects of the odors on the AB, despite giving the odors more time to be perceived. In addition, the T1 accuracy was higher for odor than no-odor trials.

As mentioned before, the previous studies by Colzato et al. (2014) involved the continuous presentation of a single odorant throughout the experimental procedure. In our studies, the presentation of odorants varied in every or every second trial. Therefore, one further potential hypothesis for the discrepancies in our results is that the continuous alternation between peppermint (proposed to be arousing) and lavender (proposed to be calming) negated the effects on arousal and concentration.

In order to determine whether our lack of significant results was due to the continuous alteration between different odorants, in Experiment 3 we included blocks in which only one odorant was presented (e.g., an entire block in which only lavender was presented). The introduction of blocks in which only one odorant was presented also increased the exposure time of each odorant, providing an even clearer test of whether an odor needs a long exposure time to influence the allocation of temporal attention. Another new component in Experiment 3 was the introduction of an “only air” block, in which no odorant was presented. This allowed for a control that was not present in Experiments 1 or 2. Finally, we also included a mixed block, which included both lavender and peppermint. The mixed block was essentially a replication of Experiment 2.

## Experiment 3

### Method

#### Subjects

Thirty-nine subjects participated in this study. The sample had a mean age of 22.10 years (*SD* = 4.2) and consisted of 35 women and four men. All subjects reported average or above average olfactory capabilities. All but five subjects reported normal or corrected-to-normal vision. As all the five subjects performed above chance and at a comparable level to other subjects (in terms of T1 and T2 detection) we decided against removing them from the data set.

#### Material, procedure, and design

Experiment 3 followed the same basic method as the other experiments. Now, there were four blocks (with 144 trials each): only air, only lavender, only peppermint, mixed block with lavender as well as peppermint. The trial procedure was exactly the same as in Experiment 2: There were alternating odor trials and no-odor trials. In the only air block, this means that in odor trials only pure air was presented. In all blocks, there were 72 odor trials and 72 no-odor trials. In the mixed block, half of the odor trials contained lavender, the other half peppermint.

Participants always began the experiment with the no-odor block. The reason for this was to avoid any influence of odors on performance in the no-odor block (i.e., to ensure it truly was a “no odor” block). The order of the remaining three blocks was counterbalanced between subjects.

The blocks with only one odorant essentially conformed to a 3 (odor: air, lavender, peppermint) × 4 (lag: 1, 3, 5, 8) × 2 (trial type: odor vs. no-odor) design. For the mixed block, the design was as follows: 2 (odor: lavender vs. peppermint) × 4 (lag: 1, 3, 5, 8) × 2 (trial type: odor vs. no-odor). In all blocks, all factors varied within subjects.

One further alteration in Experiment 3 was the introduction of the affect grid questionnaire (Russell et al., 1989), which was also used by Colzato et al. (2014). The affect grid has subjects rate their mood on a 9 × 9 grid where the vertical axis represents arousal and the horizontal axis represents valence. This affect grid was completed by subjects before each block and also after the final block of the experiment, allowing us to determine whether the specific odorants (and/or blocks) had specific effects on subjective emotionality across the experiment. Results from the affect grid questionnaire are not critical to the main purpose of the current study, and are therefore not presented in the main text (see Appendix 3 Figs. 10, 11, 12 and 13). The most important findings using the affect grid were that the lavender and peppermint odor blocks did not lead to a significant increase or decrease of reported valence or arousal, but reported valence and arousal did generally decrease with time.

#### Hypotheses

Based on the findings from Colzato et al. (2014), we would expect an interaction between odor and lag for T2|T1 accuracy. More specifically, we would predict a larger AB effect in the lavender than the peppermint condition. Alternatively, if the effects of odors are ameliorated by alternating presentation of odors, we would predict the effects to only be apparent in the single-odor blocks. Specifically, we would expect a larger AB effect in the lavender than no-odor block, while we would also expect a larger AB effect in the no-odor than the peppermint block.

### Results

**Fig. 4:**
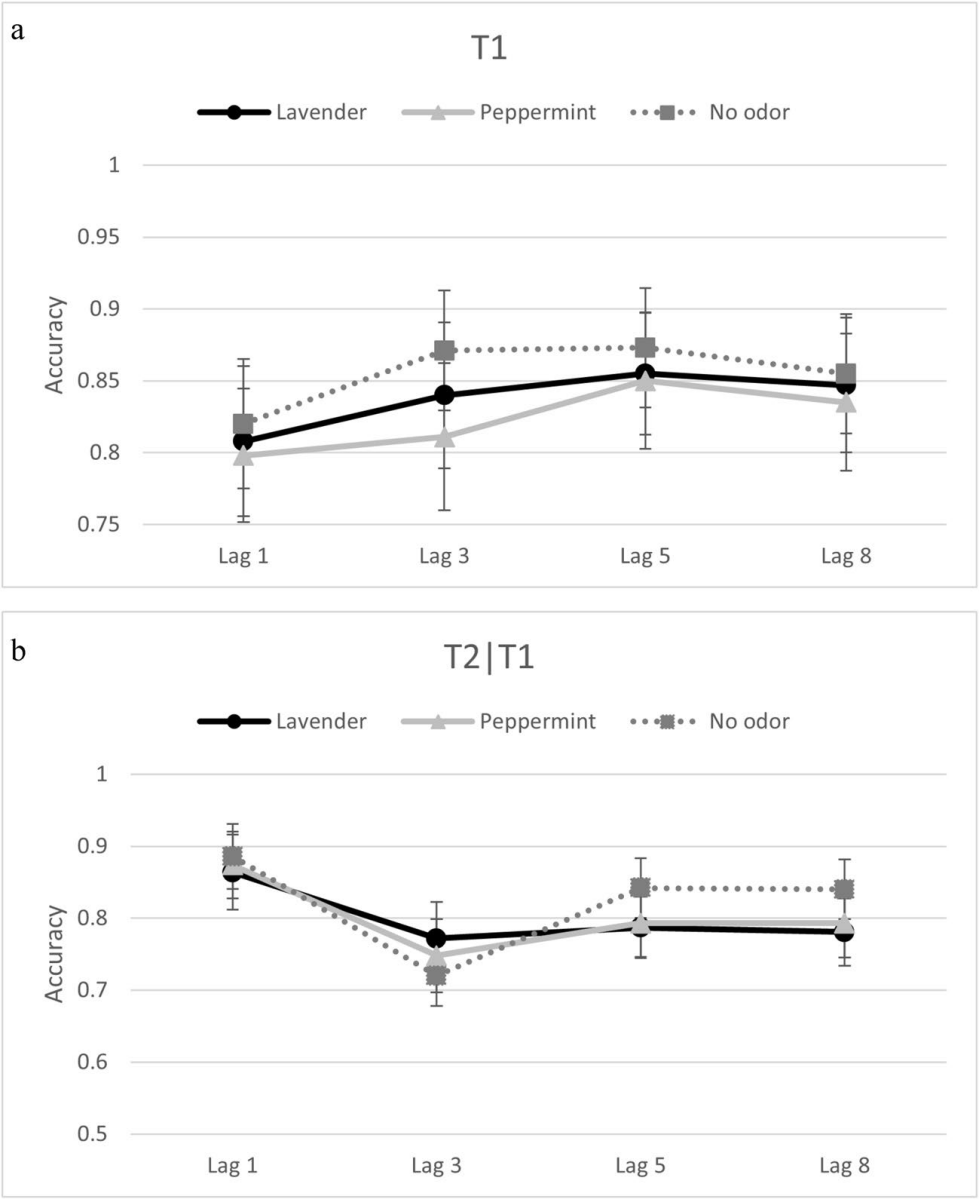
**a** Reporting accuracy for T1 as a function of odor and lag in single-odor blocks in Experiment 3. Error bars depict 95% confidence intervals. **b** Reporting accuracy for T2│T1 as a function of odor and lag in single-odor blocks in Experiment 3. Error bars depict 95% confidence intervals

**Fig. 5:**
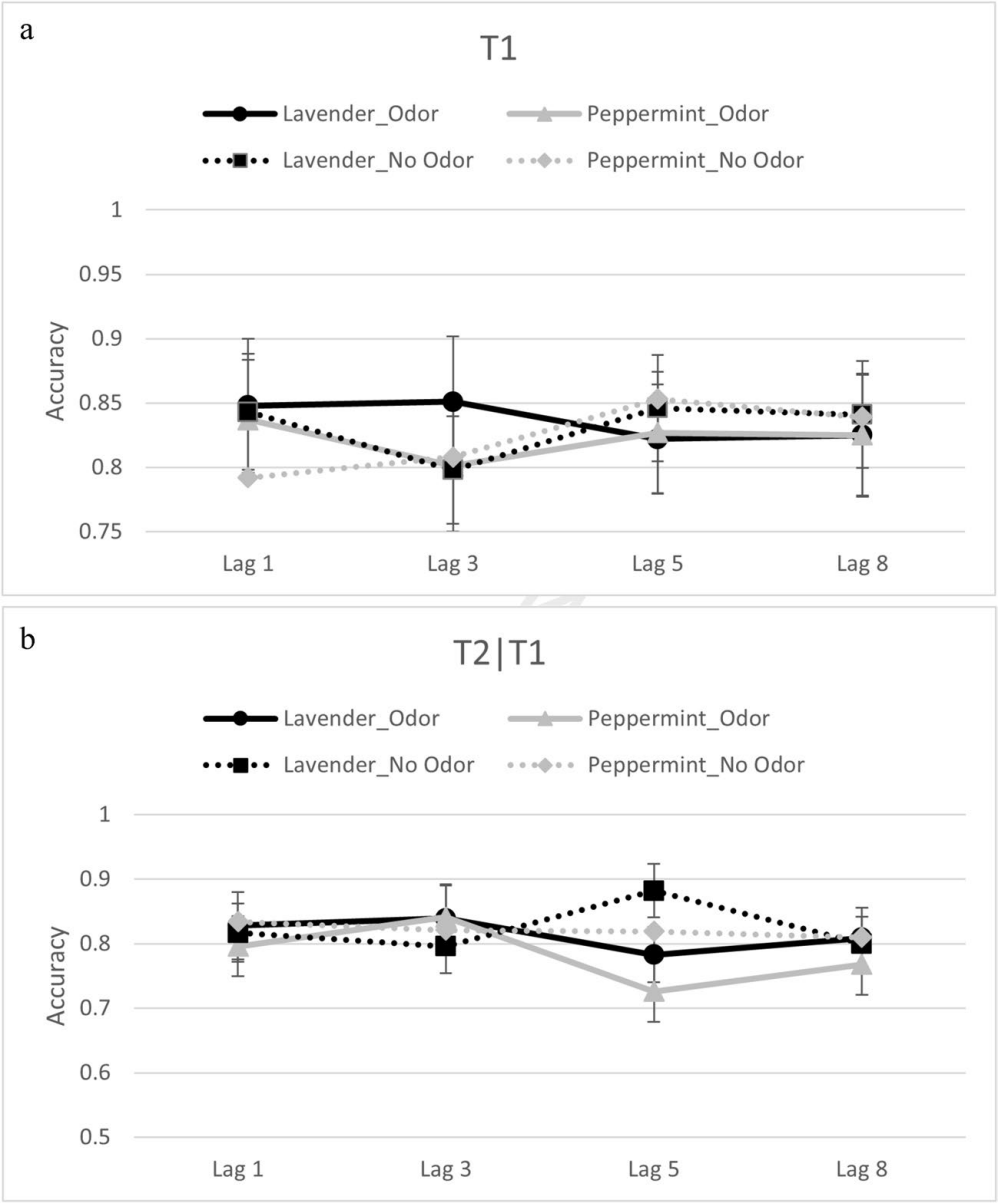
**a** Reporting accuracy for T1 as a function of odor, trial type, and lag in mixed-odor blocks in Experiment 3. Error bars depict 95% confidence intervals. **b** Reporting accuracy for T2|T1 as a function of odor, trial type, and lag in mixed-odor blocks in Experiment 3. Error bars depict 95% confidence intervals

Given the differences between the blocks with only one odorant and the mixed block (i.e., odorant changed trial-wise), we calculated ANOVAs with the factors odor, lag, and trial type, separately for the three blocks with only one odorant (i.e., only air, only peppermint, only lavender) and the mixed block (i.e., with peppermint and lavender). This also ensures direct comparison to our previous experiments. Accuracy data from T1 and T2│T1 (see Fig. 4a & b and 5a & b) were submitted to separate repeated-measures ANOVAs with the independent variables of odor, lag, and trial type, separately for the blocks with only one odorant and the mixed block.

#### Blocks with only one odorant (i.e., only air, only peppermint, only lavender)

##### T1

The ANOVA revealed a main effect of odor, *F*(2,76) = 4.93, *p* =.010, ${\eta }_{p}^{2}$ =.12. Pairwise comparisons, with Bonferroni-corrected critical *p* values =.025, revealed that T1 was detected significantly more often in the no-odor block (*M* =.86) than in the peppermint block (*M* =.82), *p* = .008, but not significantly more often than in the lavender block (*M* =.84), *p* =.058. There was no difference in T1 detection between the peppermint and lavender blocks, *p* =.163. There was also a main effect of lag, *F*(3,114) = 15.77, *p* <.001, ${\eta }_{p}^{2}$ =.29. Pairwise comparisons, with Bonferroni-corrected critical *p* values =.0167, revealed that T1 was detected less often at Lag 1 (*M* =.81), than at all other lags—that is, at Lag 3 (*M* =.84), Lag 5 (*M* =.86), or Lag 8 (*M* =.85), all *p* values <.001. Furthermore, T1 was detected less often at Lag 3 than Lag 5 (*p* =.010). Performance between Lags 3, 5, and 8 did not differ significantly, all *p* values >.05. No other main effects or interactions were significant, all *F* values < 1.22, all *p* values >.29.

##### T2│T1

The ANOVA revealed a significant main effect of lag, *F*(3,114) = 23.38, *p* < .001, ${\eta }_{p}^{2}$ = .38, which was qualified by an interaction with odor,^2^*F*(4.43,168.16) = 3.97, *p* =.003, ${\eta }_{p}^{2}$ =.10. Follow-up analyses of the interaction were carried out on the AB effects.

##### AB effect

The 3 (odor) × 2 (trial type) ANOVA revealed a main effect of odor, *F*(2,76) = 6.08, *p* =.004, ${\eta }_{p}^{2}$ =.14. A Helmert contrast revealed that the AB effect in the no-odor block (*M* = .12, 95% CI [.07, .17]) was larger than the mean AB effect for the peppermint and lavender blocks (*M* = .03), *p* = .001. The contrast also revealed that there was no difference between the peppermint (*M* = .05, 95% CI [.00, .09]) and the lavender (*M* = .02, 95% CI [−.04, .07]) blocks, *p* = .369. In accordance with the T2 | T1 analysis, the intercept was significant, *F*(1,38) = 15.20, *p* < .001, ${\eta }_{p}^{2}$ =.29, indicating an overall AB effect (independent of odor or type of trial).

#### Mixed block (with peppermint and lavender)

##### T1

The ANOVA revealed that no main effects or interactions were significant, all *F* values < 1.45, all *p* values > .23.

##### T2│T1

The ANOVA revealed that no main effects or interactions were significant, all *F* values < 2.02, all *p* values >.11.

##### AB effect

The AB effect was submitted to an odor (lavender vs. peppermint) × trial type (odor vs. no-odor) repeated-measures ANOVA. There was a significant main effect of trial type, whereby the AB effect was larger on no odor trials (*M* = .04, 95% CI [−.02, .11]) than on odor trials (*M* = −.05, 95% CI [−.10, ï.01]), *F*(1,38) = 5.63, *p* = .023, ${\eta }_{p}^{2}$ = .13. No other effects were significant, all *F* values < 2.66, all *p* values > .110.

### Discussion

Experiment 3 used long presentation durations for one odorant in the single-odor blocks, and is in this respect comparable to Colzato et al. (2014). Therefore, if the odors required longer durations to have differential effects on attention (peppermint through its arousing impact and lavender through its calming impact) this should have been reflected in the results. However, in Experiment 3 we again found no evidence for a differential modulation of the AB effects by odors. When compared to a block with no odor at all, T2 performance was equally better in the peppermint and lavender blocks.

The mixed block was essentially a replication of Experiment 2. In contrast to Experiment 2, in Experiment 3 we did not find an AB effect at all, but most important (and comparable with Experiment 2 and to our other results) we also found no difference between the peppermint and the lavender trials.

Two additional, but unanticipated, findings relate to the difference in T1 detection and the AB effect as a function of odor in the single-odor blocks. Specifically, we found higher T1 detection and a larger AB effect in the no odor block than in the odor blocks. While this may be a matter for future researchers to explore, we would caution any theoretical interpretation of this finding at the current time as it (1) was not predicted, (2) does not seem to be a hypothesis that can be clearly drawn from any theory on olfaction and cognition we are aware of, and (3) is in contrast to other findings (such as those from Colzato et al., 2014).

### Combined analyses

**Fig. 6:**
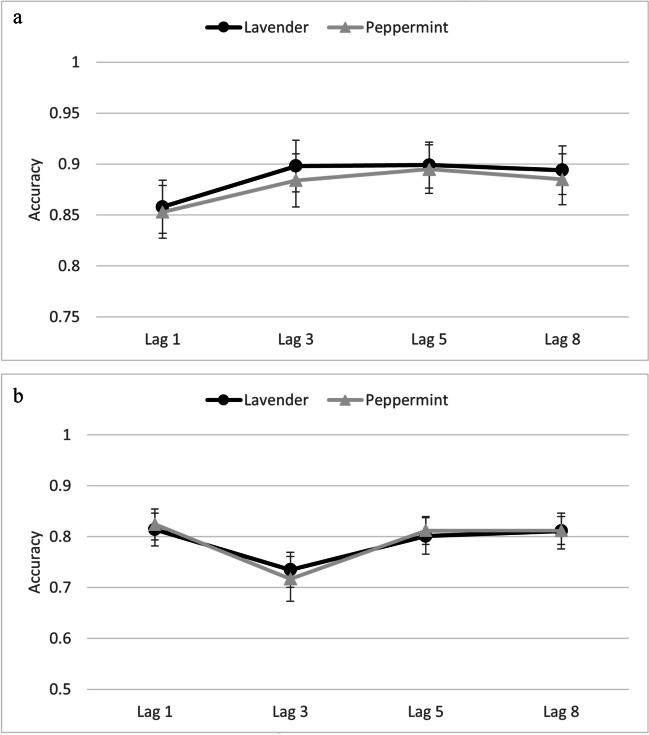
**a** Reporting accuracy for T1 as a function of odorant and lag from the analyses performed across experiments (combined analyses). Error bars depict 95% confidence intervals. **b** Reporting accuracy for T2│T1 as a function of odorant and lag from the analyses performed across experiments (combined analyses). Error bars depict 95% confidence intervals

**Fig. 7:**
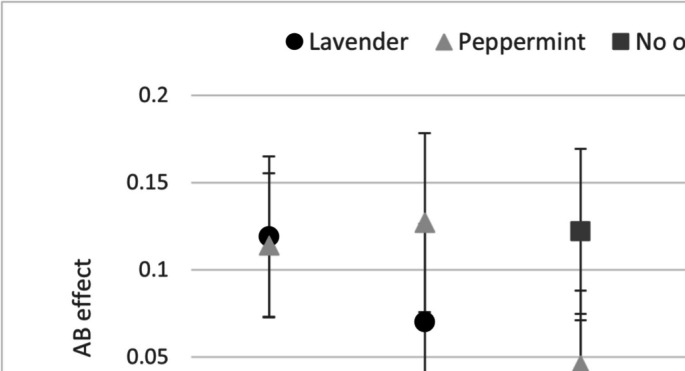
AB effect (T2|T1 Lag 5 minus Lag 3) as a function of odor across the three experiments and the combined analyses reported in the text (which excluded mixed trials). Error bars represent 95% confidence intervals. Effect sizes (*d*_z_) referring to peppermint AB effect minus lavender AB effect: Exp. 1 = −.04; Exp. 2 = .28; Exp. 3 (single) = .15; Exp. 3 (mixed) = −.15; combined across all experiments = .16

In line with our overall study goal—namely, to conceptually replicate and extend the study by Colzato et al. (2014) while using an olfactometer to allow for trial-by-trial modulation of odors on the AB task, we decided to combine the data to allow for a more powerful analysis when detecting any differences between lavender and peppermint in their effect on the AB. In order to do this, we collapsed across all variables except for odor and lag. Furthermore, we did not include data from the mixed-block from Experiment 3 due to the differences in the number of no-odor trials. Therefore, we performed a repeated measurement ANOVA with the factor experiment (Experiment 1, Experiment 2, Experiment 3) as a between-subject variable and the factors odor (peppermint, lavender) and lag (Lag 1, Lag 3, Lag 5, Lag 8) as within-subject factors, separately for T1 (see Fig. 6a) and T2│T1 accuracy (see Fig. 6b). Regarding the AB effect, we also conducted a repeated measurement ANOVA with the factors experiment (Experiment 1, Experiment 2, Experiment 3) and odor (lavender vs. peppermint) (see Fig. 7).

### Results

#### T1

The ANOVA revealed a main effect of lag, *F*(3,327) = 36.95, *p* < .001, ${\eta }_{p}^{2}$ = .25. Pairwise comparisons, with Bonferroni corrected critical *p* values = .0167, revealed that T1 was detected less often at Lag 1 (*M* = .86) than at Lag 3 (*m* = .89), Lag 5 (*M* = .90), and Lag 8 (*M* = .89), all *p* values < .001, and that detection of T1 did not differ between Lag 3, Lag 5, and Lag 8, all *p* values > .062. There was also a significant interaction between lag and experiment, *F*(6,327) = 2.41, *p* = .027, ${\eta }_{p}^{2}$ = .04. Given that we have already analyzed each experiment individually above, we did not investigate the interaction any further here. No other main effects or interactions were significant, all *F *values < 3.49, all *p* values > .064.

#### T2│T1

The ANOVA revealed a main effect of lag, *F*(3,327) = 33.22, *p* < .001, ${\eta }_{p}^{2}$ = .23. Pairwise comparisons, with Bonferroni corrected critical *p* values = .0167, revealed that detection was lower at Lag 3 (*M* = .73), than at Lag 1 (*M* = .82), Lag 5 (*M* = .81), and Lag 8 (*M* = .82), all *p *values < .001. Detection did not differ between Lag 1, Lag 5, and Lag 8, all *p *values > .348. The main effect of lag was qualified by an interaction with experiment, *F*(6,327) = 6.49, *p* < .001, ${\eta }_{p}^{2}$ = .11. Given that we have already analyzed each experiment individually above, we did not investigate the interaction any further here. No other main effects or interactions were significant; most importantly, there was no interaction of odor and lag, *F*(3,327) = 1.37, *p* = .251, ${\eta }_{p}^{2}$ = .01.

#### AB effect

The 3 (experiment) × 2 (odor) ANOVA revealed a significant main effect of Experiment, *F*(2,109) = 5.50, *p* = .005, ${\eta }_{p}^{2}$ = .09. Pairwise comparisons, with Bonferroni-corrected critical *p* values = .025, revealed that the AB effects were lower in Experiment 3 (*M* = .03) than in Experiments 1 (*M* = .12) or 2 (*M* = .10), largest *p* = .012, which did not differ from each other, *p* = .525. Importantly, the main effect of odor was not significant, *F*(1,109) = 2.50, *p* = .116, nor was the interaction between odor and experiment, *F*(2,109) = 1.08, *p* = .343. The intercept was significant, *F*(1,109) = 52.93, *p* < .001, ${\eta }_{p}^{2}$ = .33, indicating an overall AB effect (independent of odor or experiment).

**Fig. 8:**
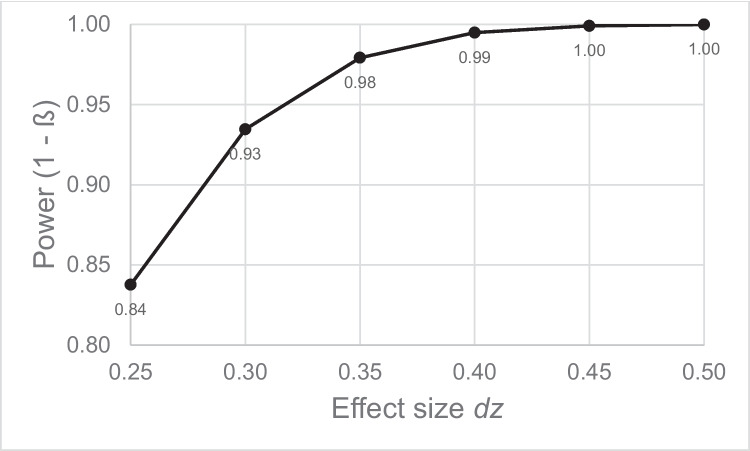
Power achieved with the combined AB effect analyses for a range of potential effect sizes ranging from *d*_z_ = .25 to *d*_z_ = .5 with a one-tailed paired-samples *t*-test, an alpha error probability of .05, and a sample size of *n* = 112

**Fig. 9:**
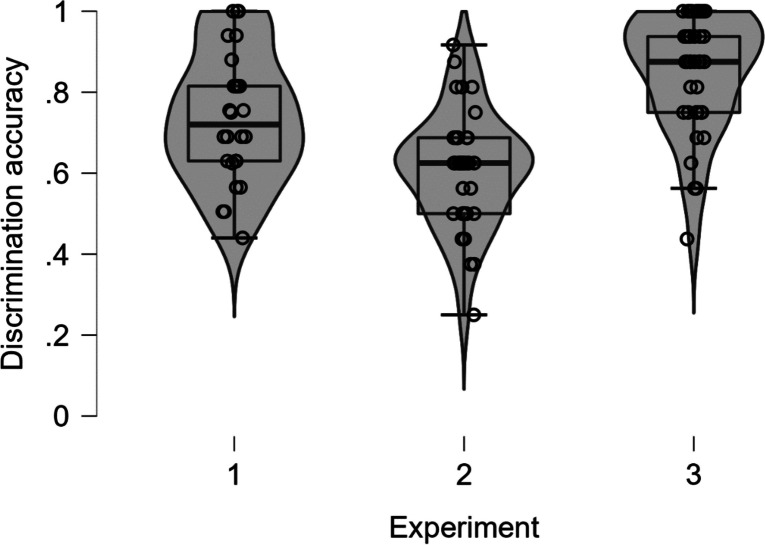
Discrimination accuracy as a function of Experiment. Box and whisker plots with distribution density. Circles represent individual data points

**Fig. 10:**
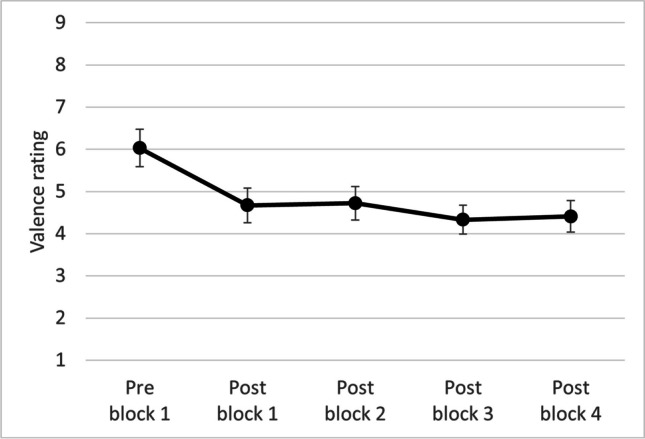
Valence ratings in Exp. 3 as a function of block when rating occurred. Error bars represent 95% confidence intervals

**Fig. 11:**
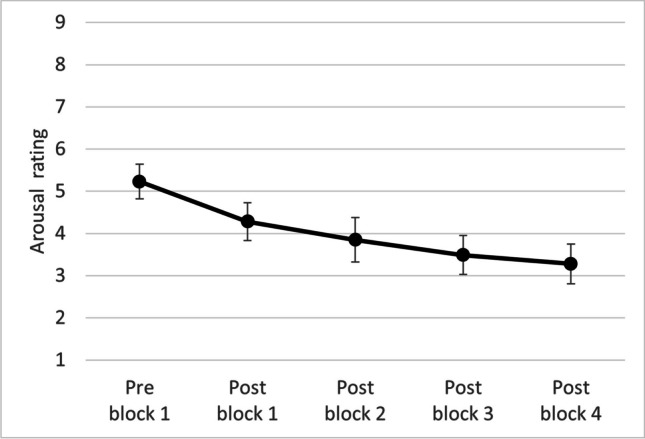
Arousal ratings in Exp. 3 as a function of block when rating occurred. Error bars represent 95% confidence intervals

**Fig. 12:**
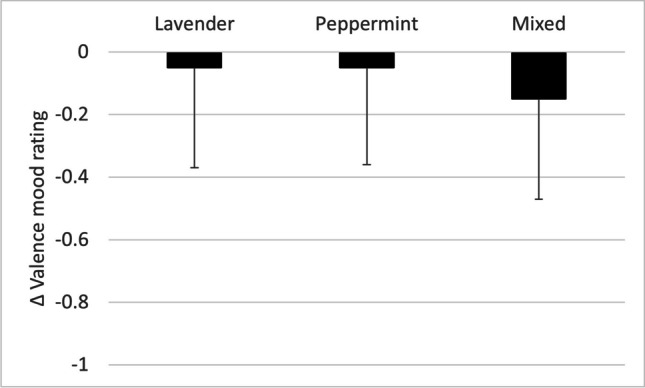
Change in (delta) valence ratings of mood as a function of odor-block in Exp. 3. Delta is calculated as the rating after the respective odor block minus the rating before the respective odor block. Error bars represent 95% confidence intervals

**Fig. 13:**
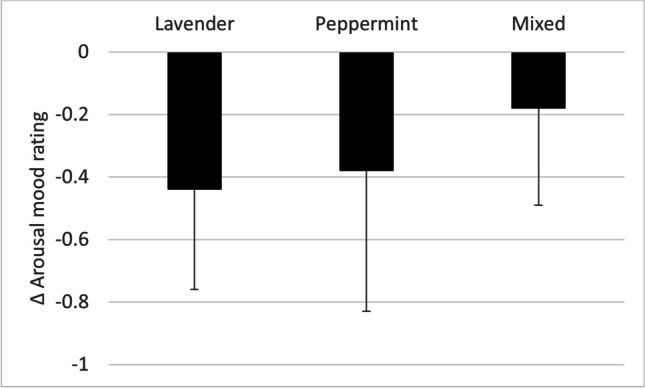
Change in (delta) arousal ratings of mood as a function of odor-block in Exp. 3. Delta is calculated as the rating after the respective odor block minus the rating before the respective odor block. Error bars represent 95% confidence intervals

We used the large sample size of this combined analysis to perform a post hoc power analysis corresponding to the difference in the AB effect between the lavender and peppermint conditions. The analysis was performed, with the aid of G*Power 3 (Faul et al., 2007), with the following parameters, which correspond to the most powerful test we could perform here: a one-tailed paired-samples *t* test, where *n* = 112 and α = .05. We set a range of effect sizes from somewhat smaller effects *d*_z_ = .25 to medium effects *d*_z_ = .5. As can be seen in Fig. 8, this power analysis makes it clear that we had achieved enough power to detect even small effects (i.e., *d*_z_ = .25) at a rate of 84% and medium effects at a rate of more than 99%.

### Discussion

The combined analyses confirmed the findings from our individual experiments. Across all experiments, we found the typical pattern for T1 detection (i.e., a generally high detection of T1 with a dip of performance when T1 was followed directly by T2—i.e., T2 at Lag 1). Moreover, these analyses confirmed the typical pattern from the AB paradigm, whereby T2|T1 detection is particularly low at lag 3 compared to lag 5, indicative of a significant AB effect. Finally, and most crucially for the current investigation, we failed to find evidence for a differential modulation of the AB effect between peppermint or lavender odorants.

## General discussion

In three experiments, we aimed to conceptually replicate the findings from experiments by Colzato et al. (2014), who reported a modulation of the AB effect by specific odorants, whereby the presentation of peppermint led to a larger AB effect than the presentation of lavender. We presented the odorants directly and on a trial-by-trial basis by means of an olfactometer, instead of ambient presentation, as was used by Colzato et al. We found the typical pattern for T1 detection as well as typical AB effects, i.e., a reduction of T2|T1 detection at lag 3 compared to lag 5. In contrast, in our three experiments and a combined analysis over all three experiments, we found no evidence for a differential modulation of the AB effect by peppermint versus lavender. The lack of modulation held, even when increasing the time in which the odor could develop its effect (either over two trials, Exp. 2, or over a whole block, Exp. 3).

Colzato et al. (2014) assumed that different arousal states, associated with peppermint versus lavender, caused differences in the AB effect, while they remained inconclusive regarding the question of whether the cause of the effect should be peppermint or lavender (see Introduction). In general, the evidence for influences of odors on arousal, specifically influences of peppermint vs. lavender on arousal, seems very weak. Colzato et al. referred to several papers (i.e., Barker et al., 2003; Basevitch et al., 2011; Ho & Spence, 2005; Ilmberger et al., 2001; Moss et al., 2003) from which they derived a stimulating influence of peppermint which increases arousal and a more focused attentional state, and a calming influence of lavender which induces relaxation and a broader attentional state. After looking more closely at the literature cited, the findings from these cited references are as follows:

Barker et al. (2003) compared performance in three tasks (memory, typing, alphabetization), once with peppermint odorant present and once without any odorant present. They found better performance in the odor condition in the gross speed, net speed, and accuracy of their typing task, as well as the alphabetization task. They found no differences between odor and no-odor condition in typing duration (what they would have expected) or the memory task. First, the results were, even for the authors, not clear and in parts not as expected. Second, it is unclear in how far the effects found are specific to peppermint (or odorants in general) as the authors did not use any further odorants. Third, there was no measure of arousal and the authors only speculate that the effect is caused by physiological changes due to peppermint, which, in turn, might increase awareness and attention leading to enhanced performance.

Basevitch et al. (2011) were interested in attention strategies during a handgrip task (i.e., a noncognitive measure of physical effort), while an adhesive strip with either peppermint (peppermint group), lavender (lavender group), or no odorant (placebo group) was placed under the participants’ nose, or there was no strip under their nose (no-odor group). Participants should squeeze the used dynamometer at their 30% max until volitional fatigue. Several times during task performance the authors measured perceived exertion, attentional strategies, perceived diversion of smell (i.e., in how far the smell subjectively diverts attention to other things besides the physical effort), pleasantness and arousal, perceived intensity of smell, and the amount of time each participant endured throughout the handgrip task. Results showed, first, that participants rated the arousal in the placebo, the peppermint and the lavender group equally. Second, odor condition did not affect the attrition rate or duration of endurance, either objectively or in the subjective rating or perceived exertion. That is, there was no difference in performance between lavender and peppermint as well as no differences between the odor and the no-odor conditions. Third, with increasing task duration, attention shifted from environmental stimuli towards emerging somatic cues and the physical task itself, but without any significant difference between odor conditions. Fourth, the authors found that participants in the lavender condition rated their perceived attentional diversion by the smell higher than the placebo or the peppermint group. Thus, it seems that participants had some expectations regarding attention associated with lavender. However, there was no effect of these expectations, much less of the actual odorants, on actual measures of attention or task performance. In addition, the only difference found was based on a difference between lavender and no odor, and could not be attributed to peppermint.

Ho and Spence (2005) investigated the influence of peppermint odorant versus clean air (and in a follow-up study cinnamon odorant or lavender odorant vs. clean air) on dual-task performance. The first task was a reaction time task: Subjects had to react with a left hand button press on number targets in a continuous RSVP stream of letter distractors. The second task was a vibrotactile compatibility task: Subjects had to lift their right toes or their right heel in response to vibrotactile stimuli presented either on front or back of their torso, while the response mapping was varied during the experiment (i.e., compatible blocks: front stimulus, lifting the toes and back stimulus, lifting the heel; incompatible blocks: front stimulus, lifting the heel and back stimulus, lifting the toes). The odors were presented alternatingly (i.e., first peppermint and then air or first air and then peppermint) and intermittently (i.e., for 35 ms at the beginning of each block and in the middle of each block, without any further olfactory presentation for the next 4 min and 40 s) by use of an olfactometer.

Ho and Spence (2005) found only one significant difference between peppermint and clean air on performance in the vibrotactile task. Subjects made fewer errors with peppermint present compared to clean air. This effect was qualified by an interaction of odor and response mapping—in the compatible (i.e., easier) condition, subjects made more errors with peppermint compared with clean air, while they made fewer errors with peppermint compared with clean air in the incompatible (i.e., more difficult) condition. But there was not a difference between peppermint and clean air in the vibrotactile condition for RTs or a difference in the RSVP task, or a difference between cinnamon or lavender and clean air in either task.

In the study by Ilmberger et al. (2001), subjects went through two blocks, the first block was with water odorant only (i.e., this was a neutral odorant, in this sense a “no-odorant” condition), the second block was with either one of the odorants of interest (experimental groups; odorants used: menthol, peppermint, cineole, ylang-ylang, jasmine) or with water again (control groups). Each block started with the presentation of the odorant (water or odorant of interest) which should be inhaled by the subjects. Thereafter, subjects rated the odorant for pleasantness, intensity, effect (from stimulating to tiring), and degree of relaxation. Then, they performed a simple reaction time task (lasting about 25 min) where they had to respond to critical stimuli by releasing a first button and pressing another button (“reaction time” = time interval from appearance of the stimulus to the release of the first button; “motor time” = time interval from releasing the first button to pressing the other button). The authors summarized the main results as follows: “comparisons between experimental groups and their respective control groups, mainly did not reach statistical significance” (p. 239). Strikingly, while response times (“motor times” as well as “reaction times”) where highly comparable in all control groups, response times of the experimental groups varied substantially across groups. In particular, motor times in Block 1 of the experimental groups were substantially smaller compared to the control groups although in Block 1 only water odor was present. Regarding the arousal measures, subjects of all conditions rated the odorant in the second block more stimulating than the odorant in the first block, without any difference between conditions and even when water was presented in both blocks. Additionally, peppermint as well as menthol (amongst others) in the second block were rated more relaxing than water in the first block. Correlations between subjective ratings and objective performance were found, thus, the authors concluded that the odor effects are mainly psychological in nature. Specifically, there were correlations between effect rating differences and response time differences in the control groups – when subjects judged the water more/less stimulatory in the second block compared to the first block, the more positive/negative response time differences were, “which clearly is a placebo effect” (p. 244).

Last but not least, Moss et al. (2003) compared three groups: lavender, rosemary, and control; the odorants were presented inconspicuously. They assessed subjective mood state (calmness, contentedness, alertness) as well as a test battery with nine cognitive tasks with 17 measures (i.e., reaction times, sensitivity, error rates) targeting attention, working memory, or long-term memory. Only six out of the 17 measures overall showed significant differences between one or two groups. The measures contributed to four factors: speed of attention, accuracy of attention, quality of memory (working memory/secondary memory), and speed of memory. Regarding quality of memory, the rosemary group outperformed the lavender group. Regarding speed of memory, the control group outperformed both the lavender and the rosemary condition. Regarding speed of attention, the control group was faster than the lavender group. There were no group differences for accuracy of attention. Regarding the mood ratings, rosemary increased alertness and contentedness while lavender and the control decreased it; the decrease in contentedness was less pronounced in lavender than in the control; there was no difference between groups regarding calmness. The authors concluded that the cognitive effects mirrored in part changes in subjective mood, and that simple changes of arousal levels are not sufficient to explain their findings. Besides the fact that only some tasks were affected by one or the other odorant—and the authors did not make any a priori hypothesis which tasks or factors should be influenced in which way—there was also no direct comparison of peppermint with lavender in this study.

Taken together, the evidence brought forward by Colzato et al. (2014; i.e., Barker et al., 2003; Basevitch et al., 2011; Ho & Spence, 2005; Ilmberger et al., 2001; Moss et al., 2003) for a differential influence of peppermint or lavender on cognitive tasks seems rather weak at best if not inconclusive, and is in part not addressing cognitive tasks at all (at least Basevitch et al., 2011). Moreover, the literature cited by Colzato et al. either tested only peppermint *or* lavender (against no odorant or other odorants; Barker et al., 2003; Ho & Spence, 2005; Ilmberger et al., 2001; Moss et al., 2003; separately tested peppermint vs. clean air and in a follow-up lavender vs. clean air) or found no relevant difference between the peppermint and lavender condition (Basevitch et al., 2011). Further, most aforementioned studies did not (directly) test whether peppermint or lavender odorants influenced arousal (Barker et al., 2003; Ho & Spence, 2005; Ilmberger et al., 2001; Moss et al., 2003) and others did not find a difference in arousal (Basevitch et al., 2011). In addition, against the background of the overinvestment hypothesis (Olivers & Nieuwenhuis, 2005), it is not clear why peppermint should change the effects in the AB task (see the Introduction). Taken together, none of the present studies showed a differential effect of lavender compared to peppermint for attention specifically or cognitive processing in general.

However, Colzato et al. (2014) reported a differential effect of lavender versus peppermint on the attentional blink effect, yet they left open how they assume that odors are able to influence the arousal state. If we consider the different ways in which odorants and/or odors can theoretically have an effect (namely, pharmacologically, psychologically or through tactile stimulation; see the Introduction), then by comparing Colzato et al.’s experiments with our own, we may be able to draw conclusions about factors more or less likely to have influenced the pattern of results in Colzato et al. Pharmacological influences can be excluded, as Colzato et al.’s experiments did not last long enough for such effects to occur. Likewise, our experiments were too short for pharmacological effects to have occurred. Additionally, the consensus from several reviews is that there is no evidence of pharmacological effects influencing human cognition and behavior in either experimental (Herz, 2009; Johnson, 2011) or clinical (Cooke & Ernst, 2000) settings, at least not due to the inhalation of odorants.

Another potential method for odorants to influence cognition is through tactile stimulation. While it is possible that lavender and peppermint differentially activate the trigeminal nerve and lead to differences in tactile stimulation, this should be the case regardless of presentation duration and manner, or the activation should be even stronger with alternating odorants compared to continuously presented ambient odorants because subjects’ sensitivity to odorants usually decreases with long-lasting or repeated presentation of the same odorant (see Ho & Spence, 2005, for this point). It then follows, that any differences between the results from Colzato et al. (2014) and our own cannot be explained by differences in tactile stimulation. We can, therefore, limit the discussion to psychological factors (e.g., mood, expectancy).

Colzato and colleagues (2014) found no evidence for a differential effect of peppermint versus lavender on affect measures. We also found no evidence that the subjects’ affect was affected differentially by peppermint vs. lavender, neither in terms of valence nor in terms of arousal (see above). Unless mood was changed only implicitly and at a non-measurable level (see Colzato et al. for this line of thought) or was changed only in Colzato et al. (implicitly and nonmeasurably) but not in our study, affect is ruled out as an explanation.

Expectations of consequences of odors have been shown to influence a range of olfactory related behaviors, from evaluative judgments of odors (de Araujo et al., 2005), and the composition of wines (Morrot et al., 2001) to the alteration of brain potentials (Lorig & Roberts, 1990). Thus, the difference between our study and Colzato et al. (2014) might be that more of their subjects had specific expectancies—based on personal past experiences—regarding the effect of peppermint or lavender. In the same vein, odors could prime concepts in a semantic network associated with them. For example, odors lead to faster processing of a word that is of a congruent than incongruent valence (Hermans et al., 1998), or a scent of a cleaning product primed cleaning behavior (Holland et al., 2005). There is also one other study on influences of odors in an AB study, also demonstrating such associative effects: Robinson et al. (2013) used olfactometer presented odorants as primes before presenting an RSVP consisting of pictures of odor-related objects and odor-unrelated objects. A decreased AB effect was reported on congruent trials (i.e., when the odor and the T2 target were semantically related; e.g., orange odorant and a picture of an orange), compared with incongruent or irrelevant trials. Referring back to our study, if the concepts associated with the odors are more or less arousing, they might differentially influence an attentional focus and, eventually, performance in an AB task. Thus, the difference between the study by Colzato et al. and ours might be that there are differences regarding idiosyncratic memories or associations (e.g., more associations of lavender and relaxation in the Colzato et al. study) leading to the differences in the results. Thus, again, there is no general effect of peppermint or lavender odors on the attentional focus, but only an individual and specific one.

One further difference between our studies and those of Colzato and colleagues (2014) relates to the breathing instructions. In our studies, we had a cue indicating when subjects should inhale (see Robinson et al., 2013, for a similar instruction). This cue was not present in the studies by Colzato and colleagues, and subjects in those studies breathed freely throughout the experiments. This difference leaves open the possibility that the different odorants led to different breathing patterns in the original study, and that the differences in breathing patterns led to differential cognitive effects (which presumably could have led to differences in the AB effect). Indeed, previous research has indicated that different breathing patterns may influence cognition (e.g., Belli et al., 2021; Heck et al., 2019). However, we believe there are several reasons to believe that the difference in breathing instruction did not lead to the differences between our study and the original study.

There is no research directly comparing how peppermint and lavender influence breathing patterns at rest. There are, however, several studies using one of the odors during sleep, in which presentation of the odorant does not appear to have influenced breathing rates (e.g., Badia et al., 1990; Carskadon & Herz, 2004) or have only short acting influences on breathing (e.g., decreased inhalation for maximum six breaths; Arzi et al., 2010). These reported (non)-influences on breathing patterns would not account for cognitive performance across an entire experiment and may be specific to direct presentation of the odorants (i.e., with an olfactometer). Furthermore, there appears to be no research showing that breathing rates influence the AB effect. Related research has, however, shown that short meditation practices (i.e., open monitoring) do not influence the size of the AB effect (see Sharpe et al., 2021, for a failure to replicate a previous study, Colzato et al., 2015). While there is a possibility that peppermint and lavender lead to differential breathing rates and this may have influenced the AB effect in the current study, more evidence would be needed before we can credibly assume that differences in breathing instructions between our experiments and those of Colzato et al. (2014) led to the differences in the AB effects.

A related point that should be considered is whether our procedure should be considered a dual task, as participants need to both attend to the RSVP and respond to the cue to inhale (i.e., by inhaling). If this was a (difficult/demanding) dual task, the overinvestment hypothesis might predict lower AB effects using our procedure. While this is an interesting methodological consideration, and it may be fair to view our procedure as a dual-task, we feel it is important to point out that the overall AB effects reported in our paper are consistent in size with those presented in previous research (e.g., Colzato et al., 2014; Robinson et al., 2013).

As we have highlighted, there were several methodological differences between our study and that of Colzato et al. (2014). The major difference was the introduction of the olfactometer in our studies to allow for direct presentation of odors, rather than the ambient presentation of odors in the study by Colzato et al. (2014). We would like to emphasize that our procedure (which was similar to that of Robinson et al., 2013) allowed us to find typical T1 reporting patterns and consistent AB effects. Furthermore, the use of the olfactometer allowed for a more controlled experimental setting, with the timing of odor presentation being able to be manipulated. This was important for empirical purposes, and allowed us to rule out certain theoretical considerations about the timing of the influences of odors on cognition. The olfactometer also allows for manipulations of odor to take place on a trial-by-trial manner (potentially making within-subjects manipulations easier to complete). We would suggest that future researchers also make use of an olfactometer for studies in which the timing of odor presentation may have an important influence and in order to ensure within-subjects manipulations can be used, given the large variation in individual responses to and preferences for different odors.

To summarize, at the moment there are two studies on the influence of peppermint versus lavender (vs. no odor) on performance in the AB task. One study (containing two experiments) used an ambient presentation of odors (Colzato et al., 2014) and our own study presented here (containing three experiments) used a direct presentation of odors by use of an olfactometer. We have now shown (1) that the differences in the (presentation) methods likely do not explain the differences in the results found, (2) that the theoretical derivation by Colzato et al., as to why peppermint and/or lavender should have a modulating effect on performance in an AB task has some gaps and is based on weak or inappropriate prior evidence, (3) that certain mechanisms (e.g., pharmacological) can be generally excluded as explanations for the pattern in Colzato et al., (4) that certain mechanisms (e.g., pharmacological or psychological) cannot be used to explain the differences found in the results, and (5) that interpersonal differences amongst the subject pools (of whatever kind, e.g., with respect to the expectations or associations they bring with them) may explain the different results, thereby ruling out a more general influence of peppermint and/or lavender.

## Conclusion

Although the idea that specific odors have a prescribed effect on human cognition is fascinating, scientific evidence for this research field, often called aromacology or aromatherapy, is sparse (for reviews, see Ali et al., 2015; Cooke & Ernst, 2000; Herz, 2009). Given the general weak evidence that specific odors have specific and consistent effects on cognition, the likewise weak evidence that peppermint and/or lavender influence arousal and, in turn, attention, and the theoretically unclear mechanisms of the potential influences of the odors, it seems unsurprising that we could not find any modulating effect of peppermint versus lavender on the AB. Based on the unclear theoretical framing and our empirical results, we conclude that lavender and peppermint do not have innate properties or systematic, stable, and universal influences on temporal attention. This goes in line with the plea for more theoretically motivated olfaction research, acknowledging the inter-individual differences associated with various odors.

## Data Availability

The data that support the findings presented in this paper are openly available on OSF (https://osf.io/5wkmg/). For anonymous peer-review, the following link can be used to access the OSF page (https://osf.io/5wkmg/?view_only=f59f9cb46e87406fbe6eb103879ff76f). Clear information concerning the materials used (e.g., the odors and olfactometer) in the experiments has been provided in the manuscript.

## Appendix 1

Discrimination rates were submitted to a One-way ANOVA with Experiment as the only independent variable. This ANOVA revealed a significant difference, *F*(2, 98) = 24.37, *p* < .001. Post-hoc comparisons, with Bonferroni corrected critical *p* values = .025, revealed that discrimination performance was better in Experiment 3 (*M* = .85, 95% CI [.80, .90]) than in Experiment 1 (*M* = .74, 95% CI [.67, .80]), *p* = .003, and that discrimination performance in Experiment 2 (*M* = .61, 95% CI [.56, .66]) was worse than in either of the other two experiments, largest *p* = .001. Furthermore, three one-sample *t* tests compared discrimination performance in each of the experiments to a guessing rate (.5). These tests revealed that performance in each of the experiments was above chance, lowest *t* = 4.76, all *p*s < .001 (see Figure 9 for a visualization of the distribution of individual data in each experiment).

## Appendix 2

Mean intensity, familiarity, and pleasantness ratings (SD in parenthesis) for peppermint and lavender across the three experiments.

**Table:**

	Experiment 1	Experiment 2	Experiment 3
Intensity			
Peppermint	5.00 (2.29)	5.58 (2.08)	6.21 (1.80)
Lavender	5.81 (2.28)	5.78 (2.21)	5.82 (1.35)
Familiarity			
Peppermint	5.97 (2.25)	6.36 (2.07)	6.79 (2.70)
Lavender	5.59 (2.37)	5.78 (2.46)	4.13 (2.06)
Pleasantness			
Peppermint	5.41 (1.74)	5.75 (1.71)	6.89 (2.18)
Lavender	5.28 (1.73)	5.31 (1.85)	4.55 (2.43)

## Appendix 3

To determine the influence of time on mood, the two dependent variables (valence and arousal) were submitted to separate repeated-measures ANOVAs, whereby report time (pre-test, post blocks 1, 2, 3, 4) was the only independent variable. Both ANOVAs revealed significant effects of time, smallest *F* = 20.45, both *p*s < .001, smallest ${\eta }_{p}^{2}$ = .35 (see Figures below for a depiction of the results).

To determine whether the specific blocks had an influence on mood, we calculated delta scores, which were equal to the ratings before a specific block subtracted from the ratings after a specific block. These delta scores (separate for valence and arousal) were submitted to separate repeated-measures ANOVAs with block (lavender, peppermint, or mixed) as the independent variable. Neither of these ANOVAs revealed a significant effect of block, largest *F*(2, 76) = .51, smallest *p* = .605 (see Figure below for a depiction of the results). The no odor block was not included, as it was always the first block and there was an obvious decrease in mood following this first block of AB trials. We interpret the decrease in mood following the no odor block to represent a general dislike for the RSVP task that was used. The task is both demanding and repetitive and it is not unexpected that mood would diminish after performing a block of trials of the task.

## Footnotes

1 Again, it should be noted here, that it is unclear how Colzato et al. (2014) calculated the AB effect, as they do not report either the formula used or the means as a function of odor and lag. Based on a visual inspection of the figures presented in their paper, our best estimate is that they used a similar calculation of the AB effect.

2 Greenhouse–Geisser correction applied.
